# Beyond the Limit: MYC Mediates Tumor Immune Escape

**DOI:** 10.3390/ph18070978

**Published:** 2025-06-29

**Authors:** Zhongyang Hong, Sitong Ming, Xin Luan, Zhe Sun, Weidong Zhang

**Affiliations:** 1 School of Pharmaceutical Science, Changchun University of Chinese Medicine, Changchun 130117, China; hzy19920605@126.com (Z.H.); mst5200915@163.com (S.M.); 2 Institute of Interdisciplinary Integrative Medicine Research, Shanghai University of Traditional Chinese Medicine, Shanghai 200120, China; luanxin@shutcm.edu.cn; 3 Department of Phytochemistry, School of Pharmacy, Second Military Medical University, Shanghai 200433, China; 4 State Key Laboratory for Quality Ensurance and Sustainable Use of Dao-di Herbs, Institute of Medicinal Plant Development, Chinese Academy of Medical Sciences and Peking Union Medical College, Beijing 100193, China

**Keywords:** MYC, immune escape, combined therapy

## Abstract

MYC is an aberrantly regulated transcription factor implicated in approximately 70% of human tumors, where it extensively modulates signaling pathways associated with cancer progression. Inactivating MYC has been shown to inhibit tumor growth and even induce sustained tumor regression across various cancer types, a phenomenon referred to as oncogene addiction. However, in vitro studies reveal that the knockout or knockdown of MYC in numerous tumor cell lines does not necessarily result in cell death, despite these tumors exhibiting MYC addiction in vivo. This discrepancy suggests that the unique tumor microenvironment in vivo may play a critical role in facilitating MYC addiction in cancer cells. MYC is also widely acknowledged for its role in mediating the immune evasion of tumor cells. Nevertheless, due to the extensive regulation of cellular gene expression by MYC and the incomplete understanding of the mechanisms underlying tumor immune escape, the precise pathways through which MYC influences tumor immune evasion remain inadequately elucidated. Recent years have seen the identification of novel tumor immune escape mechanisms, some of which have been demonstrated to be directly or indirectly regulated by MYC. For instance, MYC may contribute to immune evasion by modulating the expression of argininosuccinate synthetase 1 (ASS1), a key enzyme involved in arginine biosynthesis. Herein, in this study, we explore some novel potential mechanisms through which MYC facilitates the immune evasion of tumor cells, alongside a combined therapeutic approach targeting MYC and employing immunotherapy based on this mechanism. Furthermore, we suggest that targeting proteins interacting with MYC to modulate its expression and function may serve as an alternative strategy to direct MYC targeting, thereby expediting the clinical translation of combination therapies.

## 1. MYC

**Figure 1:**
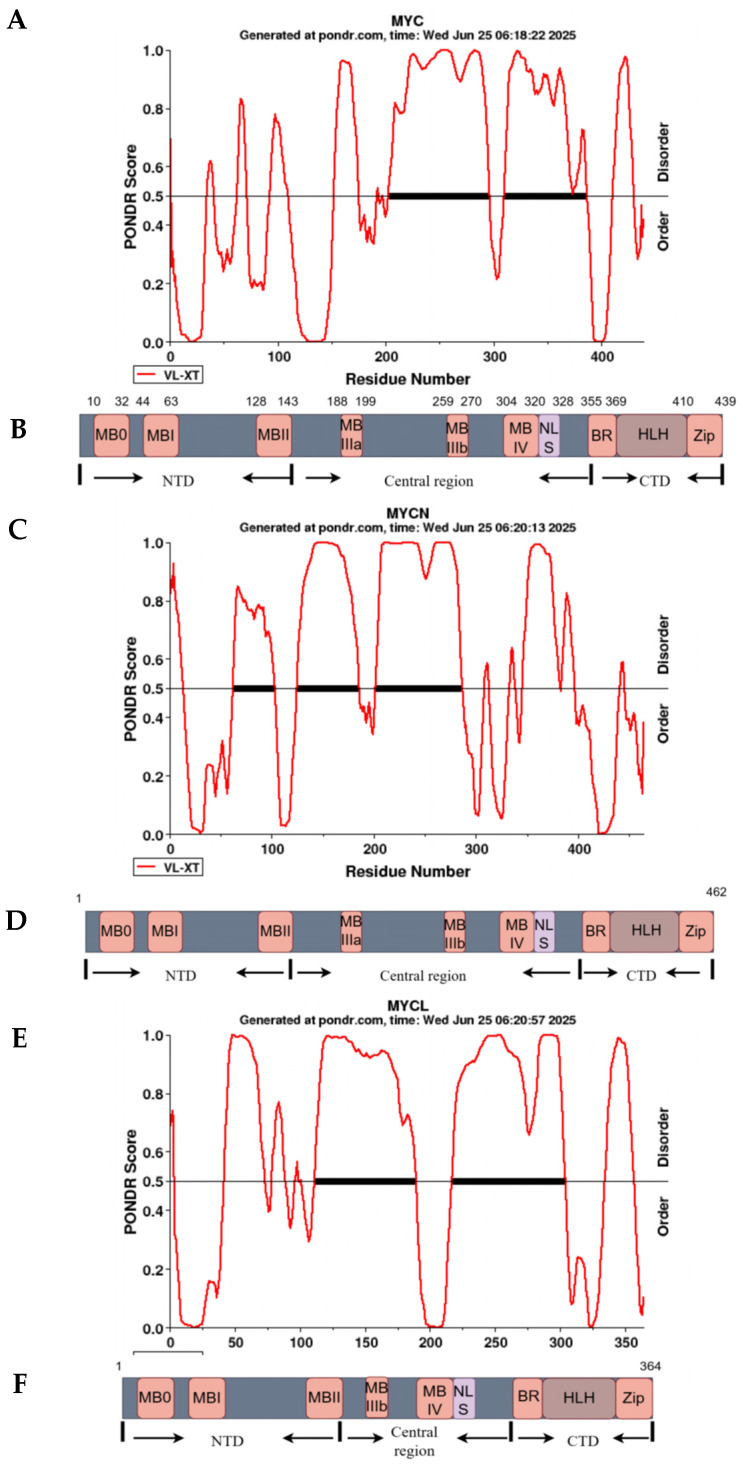
The MYC protein structure includes an (**A**) intrinsically disordered region as predicted by the (Predictor of Natural Disordered Regions (PONDR) (https://www.pondr.com/), accessed on 1 January 2025) alongside the (**B**) organization and conservation of its primary domains. (**C**) Prediction of intrinsically disordered regions in MYCN using the PONDR tool. (**D**) Structure of the primary protein domains and N-MYC conservation. (**E**) Prediction of intrinsically disordered regions in MYCL using the PONDR tool. (**F**) Structure of the primary protein domains and preservation of MYCL. The thickened straight lines represent the conservative areas.

The oncoprotein MYC is a master regulator of a diverse set of biological programmes and a transcription factor that regulates the expression of thousands of genes, either directly or indirectly [1,2]. These genes influence both cell-intrinsic biology and host immunity and the tumor microenvironment (TME) [3,4,5,6,7,8]. The MYC family comprises three oncogenes, namely, MYC, MYCN, and MYCL (Figure 1). Among them, MYC is the most widely expressed, covering almost all cancers, whereas MYCN and MYCL are expressed mainly in neuroblastoma and small cell lung cancer, respectively. Importantly, recent studies have shown that MYCN and MYCL are also expressed in other cancers, driving tumor development [9,10,11,12,13,14,15].

**Figure 2:**
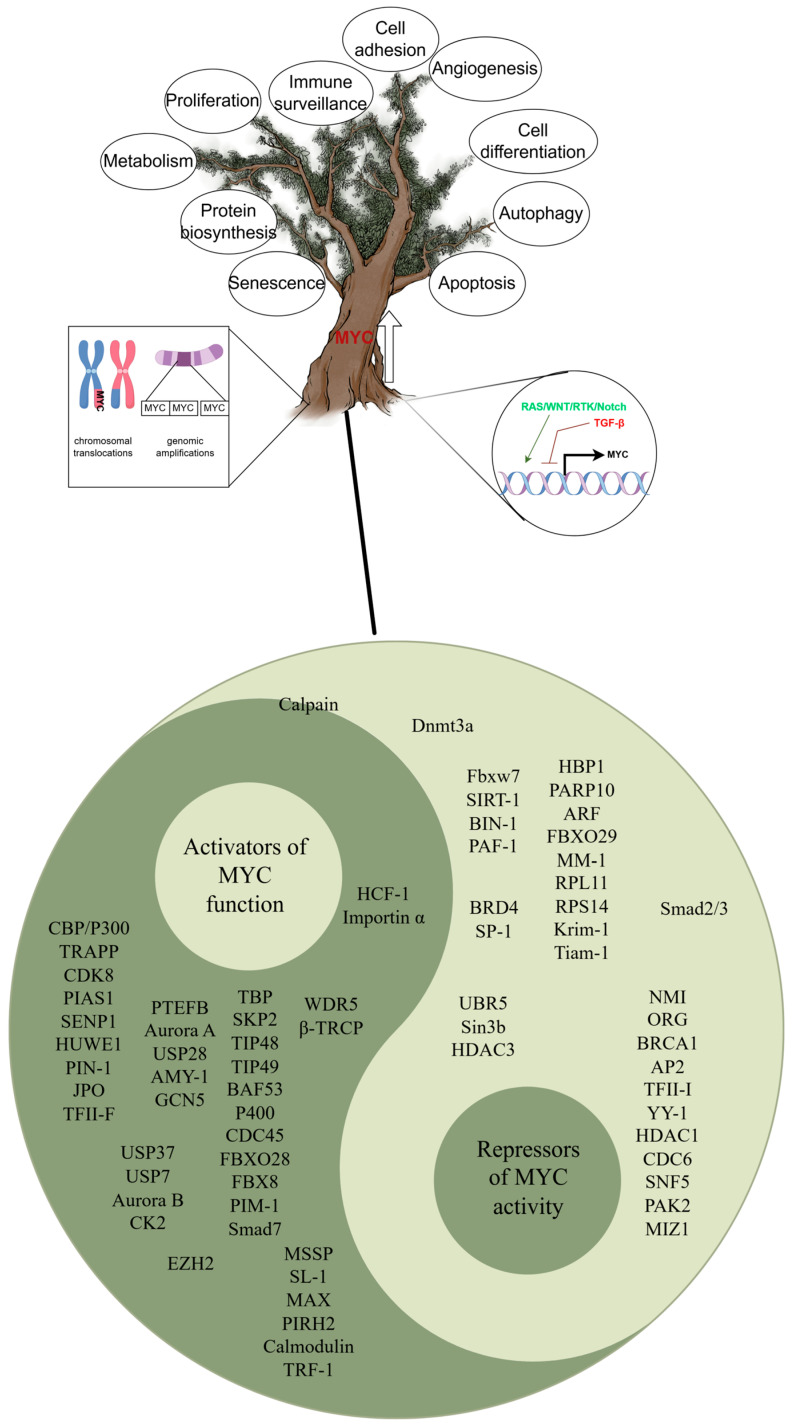
Cancer cells express high levels of the MYC protein through multiple mechanisms. Elevated levels of the MYC protein regulate various biological processes by extensively regulating the transcription of the genome.

In healthy cells, MYC is rigorously regulated to ensure that the diverse biological functions it governs are executed in a precise and coordinated manner. However, in approximately 70% of cancer cases, MYC is dysregulated, which is characterized by markedly elevated expression levels and excessive activation of its transcriptional function, enabling tumor cells to proliferate uncontrollably and potentially metastasize to nonprimary tissues. Moreover, the activation of MYC resulted in the development of an immune-suppressive, “cold” TME [16]. The mechanisms of MYC dysregulation, including gene amplification and translocation, are activated by several key signaling pathways in cancer, such as the PI3K and RAS pathways [17,18]. Moreover, the interacting proteins of MYC enable the dysregulation of MYC to be maintained and its function to be enhanced [19]. For example, by phosphorylating MYC at serine 67, Aurora B counteracts the GSK3β-induced phosphorylation at threonine 58 and the subsequent proteasomal degradation mediated by FBXW7 [20]. The G9a histone methyltransferase interacts with MYC to drive transcriptional repression and tumorigenesis [21]. In summary, various mechanisms enhance the function of MYC, allowing this powerful transcription factor to promote cancer development in numerous human cancers (Figure 2). The overexpression of MYC has been observed to have an impact on crucial biological processes, including apoptosis and DNA repair, consequently elevating the likelihood of malignant transformation [22]. In addition to its direct involvement in promoting cancer, MYC has been linked to various tumor characteristics. For example, MYC overexpression has been found to facilitate tumor angiogenesis, tumor cell invasion, and metastasis [23]. Furthermore, MYC has the ability to modulate metabolic pathways in tumor cells, enabling them to adapt to environments characterized by low oxygen levels, limited nutrients, and acidity [24,25]. Consequently, in modern cancer research, developing strategies to target MYC has gained significant attention [26].

In preclinical studies, two main strategies have been employed to modulate MYC: (1) the use of the Tet system for conditional regulation of a MYC transgene [27,28,29,30,31,32] and (2) the use of a synthetic MYC inhibitor (Omomyc [33,34,35] or MYCi361 [36]) for conditional expression. Surprisingly, in these studies, preventing MYC dysregulation not only inhibited further tumor development but also led to rapid tumor regression, a phenomenon referred to as oncogene addiction. However, in certain types of tumors, such as pancreatic cancer, knockdown of MYC does not result in tumor cell apoptosis in vitro. This may be due to the absence of the complex TME consisting of tumor cells, stromal interactions, and infiltrating immune cells. In contrast, in vivo, blocking MYC expression results in rapid tumor decrease. [32,37]. This phenomenon suggests that targeting MYC may play a critical role in regulating cell-cell interactions, particularly interactions between tumor cells and immune cells, thereby enabling tumor cells to establish a more favorable TME. Therefore, in this review, we emphasize the critical role of MYC and MYC-associated oncogenic signaling in tumor immunity and explore potential strategies for targeting MYC in conjunction with immunotherapy. We also propose that targeting MYC-interacting proteins to regulate the expression and function of MYC as an alternative strategy for direct targeting of MYC enables combination therapies to achieve clinical transformation more rapidly.

## 2. MYC and Tumor Immune Escape

Tumor immune evasion refers to the strategies employed by cancer cells to avoid detection and destruction by the host immune system, enabling their survival, proliferation, and metastasis. It is well known that these directly contribute to tumor formation and significantly influence tumor reduction when deactivated (Figure 2). Recently, a detailed pan-cancer investigation identified ENC1 (ectodermal-neural cortex 1) as a potential prognostic biomarker for TME and treatment efficacy [38]. Of note, a study has shown that MYC is capable of directly binding to the promoter region of ECN1 and exerting direct regulation over its transcriptional activity [39]. Therefore, the efficacy of targeted MYC inhibition in suppressing tumor immune escape can be evaluated by monitoring the transcriptional levels of ENC1. However, further research is essential to elucidate the systemic characteristics of MYC-targeted inhibition of tumor immune escape, thereby enabling the prediction of the prognosis of MYC-targeted therapy combined with immunotherapy across various cancer types, similar to the TGF-β signaling-related lncRNA signature identified by Wei-wei et al. [40]. Prior to this, we can systematically examine the multifaceted influence of MYC on immune cell infiltration and antitumor cytotoxicity within the TME.

### 2.1. MYC Mediates the Escape of Tumor Cells from T-Cell Immunity

In 2016, Stephanie et al. reported that MYC suppression in tumors from mice and humans lowered the levels of programmed death-ligand 1 (PD-L1) mRNA and protein [41,42]. PD-L1, also referred as CD274 or B7-H1, serves as an essential “do not find me” signal to the adaptive immune system, eliminating T-cell hostility by binding to programmed death 1 (PD-1) on T cells [43,44,45]. The PD-1 protein functions as a coinhibitory receptor and is associated with two ligands, PD-L1 and PD-L2 (B7-DC) [46,47,48,49]. Preventing PD-1 from interacting with PD-L1 strengthens immune responses in vitro and contributes to antitumor activity in preclinical experiments [50]. At present, antibodies targeting PD-1 and PD-L1 are recognized as standard care for initial cancer treatment.

Nevertheless, the response rates of just 15–50% in various solid tumors, along with high rates of progression and relapse, indicate that resistance mechanisms frequently hinder effective tumor management [51]. Transforming growth factor-β (TGF-β), a tolerogenic factor, is commonly used by cancer cells to avoid immune responses [52,53]. Growth differentiation factor 15 (GDF-15), a TGF-β superfamily produced in large quantities by various cancer types, can disrupt the antitumor immune response. Tumor-derived GDF-15 inhibits the adhesion and movement of effector T-cells into the tumor microenvironment [48]. In preclinical cancer models, blocking GDF-15 synergistically improved the effectiveness of anti-PD-1 [54]. Moreover, a clinical trial demonstrated that co-administering the GDF-15-blocking antibody visugromab with the nivolumab can counteract resistance to anti-PD-1 [51]. Following 2 weeks of anti-GDF-15 monotherapy, T-cell infiltration, growth, and activation as well as the induction of interferon-related genes and pathways within the TME [51]. At present, visugromab underwent its first human trial in a combined phase 1 and 2a study (CTL-002-001; NCT04725474). Further research on GDF-15 is necessary to explore its role, identify predictive biomarkers, and understand its mechanisms and clinical implications.

Surprisingly, GDF-15 is also subject to transcriptional regulation by MYC. MYC improves the level of GDF-15 by attaching to the E-box sequences in the GDF-15 promoter, playing a role in the positive feedback loop of GDF-15/MYC/GDF-15 [55]. Taken together, these findings suggest that MYC targeting can substantially enhance T cell-mediated tumor cell destruction.

### 2.2. MYC Mediates the Escape of Tumor Cells from Macrophage Immunity

GDF-15 was first identified as a cytokine that inhibits macrophages, known as MIC-1 [56]. Therefore, the regulation of GDF-15 by MYC also promotes the escape of tumor cells from macrophage attack. Moreover, in 2016, Stephanie et al. also reported that reducing MYC in mouse tumors and human cancer cells led to decreased levels of CD47 mRNA and protein [41,42]. Chromatin immunoprecipitation sequencing (ChIP-Seq) demonstrated that MYC binds to the promoters of CD47 and PD-L1, mechanistically [33,34]. CD47 is a critical “do not eat me” signal to the innate immune system as well as a regulator of the adaptive immune response [57,58]. CD47 on the tumor cell surface interacts with inhibitory macrophage receptor signal regulatory protein α (SIRPα) to send a signal that inhibits phagocytosis, preventing cancer cells from being cleared by macrophages [48,49]. Moreover, CD47 promotes tumor progression in various malignancies by interacting with thrombospondin-1 (TSP-1) [59].

**Figure 3:**
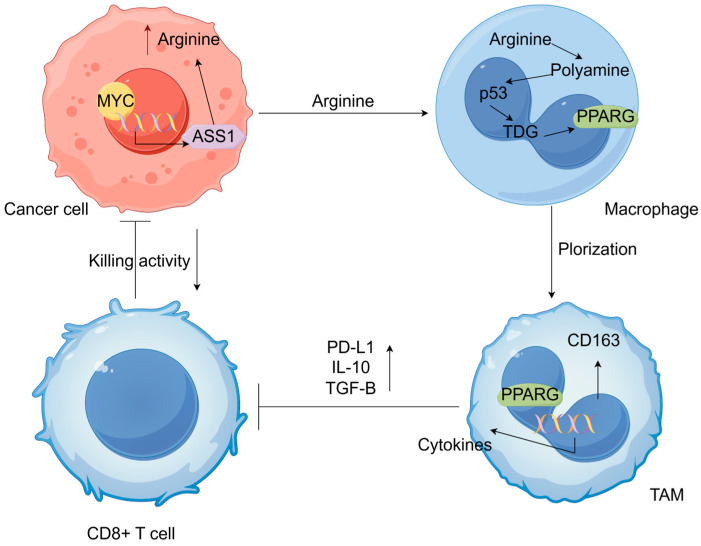
In cancer cells, MYC upregulates ASS1 expression, enhancing arginine biosynthesis and elevating its levels in the TME. This process effectively suppresses the antitumor function of CD8+ T cells, outweighing any potential arginine-mediated enhancement of their cytotoxic activity. Mechanistically, arginine is metabolized into polyamines within TAMs, which then promote their protumor polarization through TDG (thymine DNA glycosylase)-dependent DNA demethylation—a pathway modulated by p53 signaling. Arrows denote upregulation or an increase in expression levels, whereas other types of indicator lines represent inhibition.

In addition to directly regulating the expression of CD47, MYC may further help tumor cells escape phagocytosis by regulating the expression of argininosuccinate synthetase 1 (ASS1), a key enzyme in arginine biosynthesis [60] (Figure 3). Arginine is a semi-essential amino acid that the body can produce internally, but it often needs to be supplemented from external sources, especially during growth, wound recovery, or in tumors with reduced arginine production. It plays a role in protein synthesis, cell growth, and immune system regulation [61]. Keshet et al. demonstrated that ASS1 expression is induced by MYC under glucose deprivation [62]. Another study indicates that MYC transcriptionally regulates ASS1 reactivation through the upstream Gas6-Axl tyrosine kinase (RTK) signaling pathway [63]. Moreover, our RNA-seq data obtained from the pancreatic cancer cell line Mia-Paca2 revealed a substantial reduction in ASS1 mRNA levels following MYC knockdown. ASS1 downregulation promotes cell proliferation in various tumors; its expression correlates with poor prognosis in several common cancer subsets [62]. Decreasing ASS1 allows more aspartate to be used in pyrimidine synthesis, thereby supporting cell proliferation [64]. However, increased pyrimidine levels result in a mutational bias towards pyrimidine, which is connected to elevated hydrophobic-immunogenic antigens and a heightened response to immunotherapy [65]. In a recent study, Yinghua et al. highlighted the significant role of metabolic interactions between cancer cells and macrophages in promoting arginine-driven breast cancer progression [66]. Taken together, targeting MYC significantly enhances macrophage-mediated tumor cell destruction.

### 2.3. MYC May Mediate Immune Escape of Tumor Cells by Inducing Neutrophil Infiltration

Neutrophils that are pathologically activated, known as myeloid-derived suppressor cells (PMN-MDSCs), play a significant role in suppressing antitumor immunity [67,68,69]. It is known that MYC promotes neutrophil infiltration in the external tumor environment [70]. For example, in a zebrafish liver tumor model study, the phenomenon of MYC overexpression, which incurred an increase in liver-infiltrated neutrophils, was successfully observed [70]. Moreover, MYCN also modulates infiltration of neutrophils through attenuation of NF-kappaB signaling by activating NDRG1/Cap43 pathway [71]. Recent research has shown that neutrophils physically interact with tumor cells to form a signaling niche that promotes breast cancer aggressiveness [72]. A separate study demonstrated that co-culturing renal cell carcinoma (RCC) cells with neutrophils can specifically impact MYC signaling, thereby promoting RCC cell proliferation [73]. These findings suggest that MYC facilitates tumor development by recruiting neutrophils to infiltrate the TME. Nevertheless, the question remains whether neutrophils within the TME contribute to the immune evasion of tumor cells. This topic will be briefly explored in the subsequent discussion.

Rina et al. demonstrated that PMN-MDSCs in the TME die spontaneously via ferroptosis [74]. Ferroptosis is a type of regulated cell death that is not apoptotic, initiated by the imbalance of redox regulatory processes, leading to extensive peroxidation of polyunsaturated phospholipids. In addition, by lowering PMN-MDSCs, ferroptosis promotes the release of oxygenated lipids and curtails T-cell activity in mice and humans [74]. In mouse models of spontaneous tumor development, interfering with IL-1β+ CD4+ T cells or IL-1R1+ neutrophils interrupted communication, reduced neutrophil ferroptosis, boosted antitumor immunity, and countered chemoresistance [75]. Therefore, inhibiting the ferroptosis of neutrophils is beneficial for immune cells to kill tumor cells. Taken together, neutrophils in the TME may promote immune escape of tumor cells through spontaneous ferroptosis.

Of note, the regulation of ferroptosis by MYC has been widely reported. For example, MYC enhances cellular antioxidant capacity and suppresses ferroptosis in cancer stem cells by activating CPT1A expression, which in turn activates the NRF2/GPX4 system and reduces phospholipid polyunsaturated fatty acids via ACSL4 downregulation [76]. Moreover, MYC directly downregulates the expression of NCOA4, thereby inhibiting ferroptosis, promoting ovarian cancer cells’ malignant phenotype and immune evasion by suppressing ferritin autophagy [77]. Furthermore, MYC reduces the inhibition of pan-death, including ferroptosis, in colorectal cancer tumor cells by transcriptionally regulating NFS1, thereby decreasing their chemosensitivity [78]. These studies have shown that MYC inhibits ferroptosis in tumor cells and is an important cause of ferroptosis resistance in tumors [79,80]. However, a limited number of studies have also suggested that MYC plays a role in promoting ferroptosis. For example, Hamed et al. demonstrated that MYCN mediates cysteine addiction and sensitizes neuroblastoma to ferroptosis [81]. Moreover, the upregulation of ACSL4 by MYC, driven by oncogenic RTKs, increases cancer cells’ sensitivity to ferroptosis [82]. The variations in the regulatory effect of MYC on ferroptosis across different tumor cells may be attributed to the distinct characteristics of oncogenes and the heterogeneity of the TME. In treatment-naive high-grade serous ovarian cancer (HGSOC), investigating TME cell composition, DNA copy number, mutations, and gene expression demonstrated that immune cell exclusion correlated with the amplification of MYC target genes [83]. However, unfortunately, no studies have reported whether MYC exerts a regulatory effect on ferroptosis in neutrophils. Moreover, tumor-associated neutrophils (TANs) are heterogeneous; thus, their roles in tumor development could vary depending on the cancer type [84]. Therefore, investigating the role of MYC in regulating ferroptosis in neutrophils across different cancers may represent a significant research gap that warrants further exploration. Specific experiments to test if MYC regulates key ferroptosis drivers (GPX4, ACSL4, etc.) specifically in PMN-MDSCs/TANs are necessary and meaningful. To summarize, these findings indicate that targeting MYC to attenuate neutrophil infiltration within the TME can not only suppress tumor cell proliferation and invasion but may also contribute to inhibiting tumor immune escape.

### 2.4. MYC Mediates the Escape of Tumor Cells from Natural Killer (NK) Cells Immunity

NK cells, which have cytotoxic functions similar to those of CD8+ T cells, are another powerful weapon against tumors. Deactivating MYC in existing adenocarcinomas immediately reverses stromal changes and causes tumor regression, which is not dependent on CD4+CD8+ T cells but heavily relies on the return of NK cells [85]. Research has further revealed that MYC-dependent tumor progression requires IL-23 signaling to NK cells, thereby promoting the exclusion of NK cells from the TME [85]. IL-23 is a proinflammatory cytokine that hinders wound healing and promotes tumor growth in various tissues [86]. It also strongly inhibits innate immunity, particularly affecting NK cells [87]. The absence of IL-23 in mice led to resistance against experimental tumor metastases in three models, where host NK cells were responsible for disease control [87]. IL-23 has been shown to be positively regulated by MYC [88,89]. For instance, the increased expression of IL-23 observed in Mst1-knockdown dendritic cells (DCs) was effectively downregulated by MYC inhibitors [89]. Moreover, inactivation of MYC also increases the level of NKG2DL in N-type small cell lung cancer (SCLC-N) cells, resulting in NK cell recruitment [90]. Originally found in NK cells, NKG2D identifies ligands that are elevated on cancer cells [91]. MYC-MIZ1 repressive complexes bind directly to the promoters of the genes encoding the type I IFN regulators to inhibit the expression of these genes, such as STAT1, STAT2, IRF5, and IRF7 [92]. Moreover, derepression of IFN regulator genes facilitates the infiltration of pancreatic tumors by B and natural killer cells, resulting in increased survival [92]. Taken together, MYC in tumor cells appears to reduce the number of NK cells in the TME through a number of mechanisms.

## 3. Strategies Combining the Targeting of MYC and Immunotherapy

### 3.1. Inhibition of MYC Combined with Cell Therapy

**Figure 4:**
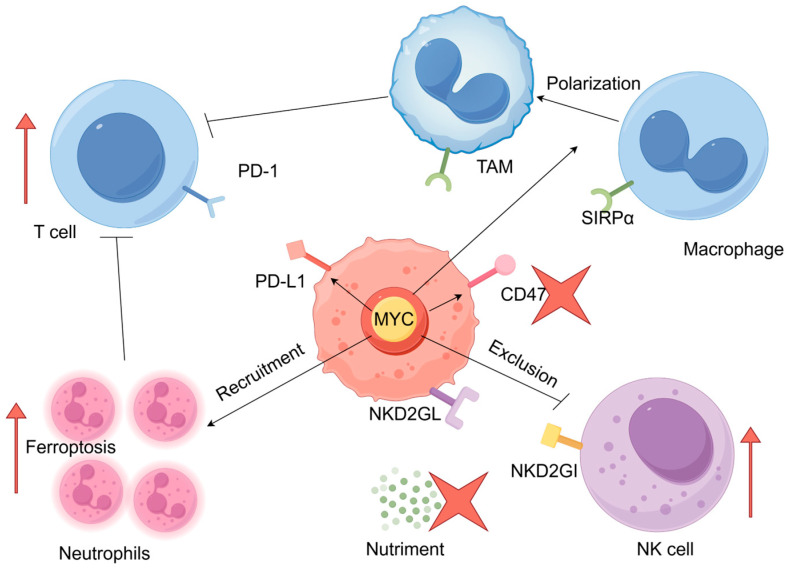
The impact of MYC on the tumor immune microenvironment and potential combinatory treatment strategies. The red arrow signifies the necessity of enhancing the process, while the four-leaf clover symbolizes the inhibition of the process. Of note, the red arrow points to the neutrophil, indicating an increased level of ferroptosis in this cell type.

Previously, we discussed the regulatory role of MYC in tumor immune escape. On the basis of these studies, we discuss possible strategies for targeting MYC in combination with immunotherapy (Figure 4). First, MYC has been found to upregulate the expression of the immune checkpoint PD-L1, thus leading to T-cell exhaustion [41,85,93,94,95]. Of note, the inhibition of MYC results in the expression of PD-L1 decreasing significantly, thereby increasing the number of surviving T cells in the TME. In addition, targeting MYC can significantly increase the proportion of CD8+ T cells, thereby enhancing the killing effect of T cells on tumor cells [36]. Therefore, targeting MYC can be combined with chimeric antigen receptor-modified T (CAR-T) cell therapy to replenish exhausted T cells in the TME. CAR-T cell therapy has been highly successful in treating blood cancers, but it is less effective against solid tumors, partly because of CAR-T cell exhaustion in the solid tumor microenvironment [96]. Moreover, genetic downregulation of ID3 and SOX4 expression can improve the efficacy of CAR-T cell therapy in solid tumors by preventing or delaying CAR-T cell dysfunction [96]. ID3 and SOX4 are positively regulated by MYC [97,98]. Therefore, inhibition of MYC in cancer cells can promote the therapeutic effect of CAR-T cells on solid tumors.

In addition to CAR-T cells, CAR-NK cells have become a research hotspot since 2025 because of their “off-the-shelf” characteristics. It has been confirmed in multiple mouse models that CAR/IL-15 NK cells from optimal cord blood units (CBUs) in vivo have superior antitumor activity [99]. Jianhua et al. demonstrated that neoleukin-2/15-armored CAR-NK cells sustain superior therapeutic efficacy in solid tumors via MYC/NRF1 activation [100]. The findings indicate that MYC is essential for Neo-2/15-driven ATP generation by CAR-NK cells in the TME [100]. Moreover, Daher et al. reported a strategy that combines the targeting of the cytokine-inducible Src homology 2-containing (CIS) protein, a key negative regulator of interleukin 15 (IL-15) signaling, with fourth-generation “armored” CAR engineering of cord blood-derived NK cells [98]. The combined strategy enhances NK cell effectiveness by promoting the Akt/mTORC1 pathway and MYC signaling, which increases aerobic glycolysis [101]. Moreover, through transcriptome and chromatin immunoprecipitation analysis, MYC was identified as a direct downstream target of XBP1, playing a role in NK cell proliferation regulation [102]. Taken together, these findings suggest that enhancing MYC signaling within CAR-NK cells may increase their therapeutic efficacy.

### 3.2. Inhibition of MYC Combined with Monoclonal Antibodies

In addition to regulating PD-L1, MYC has also been identified as a transcription factor for GDF-15 [55]. Targeting GDF-15 has been shown to overcome the resistance associated with PD-1/PD-L1 monoclonal antibody therapy [51]. Consequently, targeting MYC in combination with PD-1/PD-L1 monoclonal antibodies may serve as a promising therapeutic strategy. The application of PD1/PD-L1 monoclonal antibodies in combination with MYC inhibition not only aids in overcoming drug resistance but also decreases the required dosage of PD1/PD-L1 monoclonal antibodies. Moreover, antibodies targeting the immune checkpoint molecules PD-1, PD-L1, and cytotoxic T-lymphocyte antigen 4 (CTLA-4), administered alone or in combination with chemotherapy, are the standard of care in most patients with metastatic non-small cell lung cancers and other cancers [103,104]. Of note, dual blockade immunotherapy targeting PD-1/PD-L1 and CTLA-4 has achieved remarkable success in various cancers [105,106,107,108]. In light of the positive regulatory effect of MYC on PD-L1, inhibition of MYC in combination with CTLA-4 monoclonal antibodies may serve as an optional therapeutic strategy.

As elucidated in the preceding sections, MYC-driven macrophage immune evasion primarily involves the transcriptional upregulation of CD47, which collectively suppresses phagocytic activity and creates an immunosuppressive microenvironment [51]. The dual blockade of galectin-3 (Gal-3) and CD47 synergistically suppressed tumor growth, increased phagocytosis, repolarized macrophages, and increased T-cell immune responses [106]. However, depletion of Gal-3 reduces the expression of CD47 through inhibition of the binding of MYC to the CD47 promoter [109]. Therefore, targeting MYC in combination with CD47 monoclonal antibodies may also serve as a promising therapeutic strategy.

### 3.3. Inhibition of MYC Combined with Adjustment for Nutritional Intake

Cancer cells rewire metabolism to favor the generation of specialized metabolites that support tumor growth and reshape the tumor microenvironment [110,111]. A recent study reported that a lysine-restricted (LR) diet synergized with MYC inhibition or anti-PD-1 therapy to slow tumor growth [112]. Mechanically, LR diet enhances IFN signalling and functional CD8+ T cell infiltration [112]. This study successfully established a connection between targeting MYC and dietary and nutritional interventions. However, the efficacy of the LR diet has only been demonstrated at the animal experimentation stage, and significant further research is required before this strategy can be translated into clinical application. Moreover, whether restricting other single amino acids may similarly facilitate tumor immune escape. For example, breast cancer cells promote tumor-associated macrophage polarization via arginine secretion, thereby suppressing CD8+ T cell cytotoxicity against tumors [66]. Therefore, dietary arginine restriction may enhance immune-mediated tumor cell killing. In light of the regulatory influence of MYC on ASS1, targeting MYC and inhibiting arginine uptake may offer greater therapeutic benefits for breast cancer patients [66]. Moreover, Cao et al. suggested that tumoral SLC6A6-mediated taurine deficiency promotes immune evasion and that taurine supplementation reinvigorates exhausted CD8+ T cells and increases the efficacy of cancer therapies [113]. SLC6A6 may be directly or indirectly regulated by MYC [113,114]. Therefore, targeting MYC and taurine supplementation may enhance the tumor-killing capacity of immune cells. Beyond amino acids, vitamins have garnered increasing attention for their multifaceted roles in cancer treatment, with accumulating evidence supporting their potential in prevention, adjuvant therapy, and immunomodulation. A recent study revealed that cancer cells exploit circulating lipoproteins to acquire α-tocopherol (the predominant form of vitamin E in human lipoproteins), thereby mitigating lipid peroxidation toxicity and enhancing resistance to ferroptosis [115]. Moreover, A clinical study investigating breast cancer has demonstrated that supplementing with a low dose of vitamin D can enhance the efficacy of chemotherapy in breast cancer patients. Specifically, patients who received a daily supplementation of 2000 IU of vitamin D for six months showed a pathological complete response rate of 43% in the vitamin D supplementation group [116]. However, whether the selective uptake of vitamin components and the targeting of MYC exhibit a synergistic effect has not yet been systematically investigated to date. This area of research may hold significant potential for further exploration. Taken together, strategic modulation of MYC signaling pathways coupled with tailored nutritional interventions may constitute a multimodal paradigm with substantial translational potential for cancer therapeutics. Of note, given the substantial complexity and heterogeneity of the TME, whether restricting a single nutrient intake suffices to elicit desired therapeutic outcomes or whether combined targeting of MYC enhances efficacy requires rigorous experimental validation.

## 4. Future Directions

Insights into the regulatory role of MYC in the host immune response hold significant practical implications for the development of therapeutics and the strategic selection of therapies. Targeting MYC, either alone or in conjunction with immunotherapy, is anticipated to offer a curative approach for a majority of cancers. However, as of now, no direct MYC inhibitors have received approval for clinical use. Consequently, the development of effective and safe strategies to suppress MYC expression in cancer cells remains a critical challenge. This necessitates innovative approaches to mitigate its pleiotropic oncogenic functions while minimizing off-target effects. The development of targeted therapies aimed at disrupting the oncogenic functions of MYC has become a central focus in cancer research, with particular emphasis on modulating MYC-interacting protein networks. This strategy is exemplified by recent advancements in destabilizing MYC-MAX heterodimers through engineered peptides, such as Omomyc, and proteolysis-targeting chimeras (PROTACs), which induce MYC degradation [117,118]. Moreover, strategies targeting MYC transcriptional regulation, translational control, protein stability, and transcriptional activity have now been proposed (Table 1). In 2020, Lourenço C et al. systematically reviewed the therapeutic strategies targeting MYC, which we only briefly touch upon in this context [19].

Notably, MYC-mediated immune evasion is characterized by tumor type-specific mechanistic heterogeneity, necessitating tailored combinatorial immunotherapies that target distinct immune suppressive pathways. Therefore, the identification of biomarkers capable of predicting prevalent MYC-driven immune escape mechanisms in individual patients and tumor types is essential to guide personalized combinatorial therapeutic strategies. Of note, owing to MYC’s pleiotropic regulation of cellular processes, targeting MYC represents a promising strategy to overcome immunotherapy resistance [119,120]. Therefore, directly targeting MYC remains the most critical challenge to overcome in current oncology therapeutics. Moreover, the development of additional preclinical models is imperative to rigorously evaluate the efficacy of combinatorial therapeutic regimens and derive clinically actionable treatment strategies. Our strategy should first assess whether MYC targeting modulates immune cell infiltration in the TME or enhances the antitumor activity of immune cells. Based on these findings, we can then rationally select combination therapies. Bioinformatics analysis may further guide the selection of appropriate preclinical models to validate these hypotheses. In summary, as our understanding of MYC biology has increased and immunotherapeutic strategies, such as immune checkpoint blockade, have demonstrated clinical efficacy in treating hematologic malignancies, we have positive hopes for the future.

**Table 1:** Main strategies targeting MYC in cancer therapy.

Strategies	Representative Target	Promising Therapeutic Agents	References
MYC-MAX dimerization disruption	MYC-MAX	Omomyc	[98]
		KSI-3716	[121]
		MYCi361/MYCi975	[36]
		VPC-70067	[122]
		10058-F4	[123]
		IIA6B17	[124]
		10074-G5	[125]
		MYCMI-6	[126]
		H1 peptide	[127]
		MI1-PD	[128]
		KJ-Pyr-9	[129]
		Mycro3	[130]
Disrupt the binding of MYC-DNA	MYC-DNA	ME47	[131]
Transcriptional and translational suppression	CDK7	SNS-032	[132]
	CDK9	AZD4573	[133]
	BRD4	JQ-1	[134]
Targeting interacting proteins	AURKB	AZD-1152	[19,135]
	WDR5	C6	[136]
	EZH2	Tazemetostat	[137]
Targeting the upstream signaling pathways	RAS	RMC-7977	[138]

## Data Availability

Not Applicable.
